# Decoding LAG3 activation: ubiquitination liberates immunosuppressive motif to license checkpoint function

**DOI:** 10.1186/s43556-025-00344-w

**Published:** 2025-11-04

**Authors:** Tong Zhang, Feng Xie, Fangfang Zhou

**Affiliations:** 1 Department of Ultrasound, the First Affiliated Hospital, Suzhou Medical College, Soochow University, Suzhou, Jiangsu China; 2 Life Sciences Institute and State Key Laboratory of Transvascular Implantation Devices of the Second Affiliated Hospital of Zhejiang University School of Medicine, Zhejiang University, Hangzhou, 310058 China; 3 The Institutes of Biology and Medical Sciences, Suzhou Medical College, Soochow University, Suzhou, China

A recent study published in *Cell* reveals how ligand binding activates lymphocyte activation gene 3(LAG3) [1]. They found this interaction triggers rapid non-degradative polyubiquitination of LAG3, releasing its immunosuppressive signaling motif from membrane sequestration (Fig. 1) [1]. This discovery not only settles enduring debates surrounding LAG3 activation but also pinpoints the co-expression of LAG3 and CBL as a possible biomarker to forecast patient reactions to LAG3-targeted immunotherapy.

**Fig.1:**
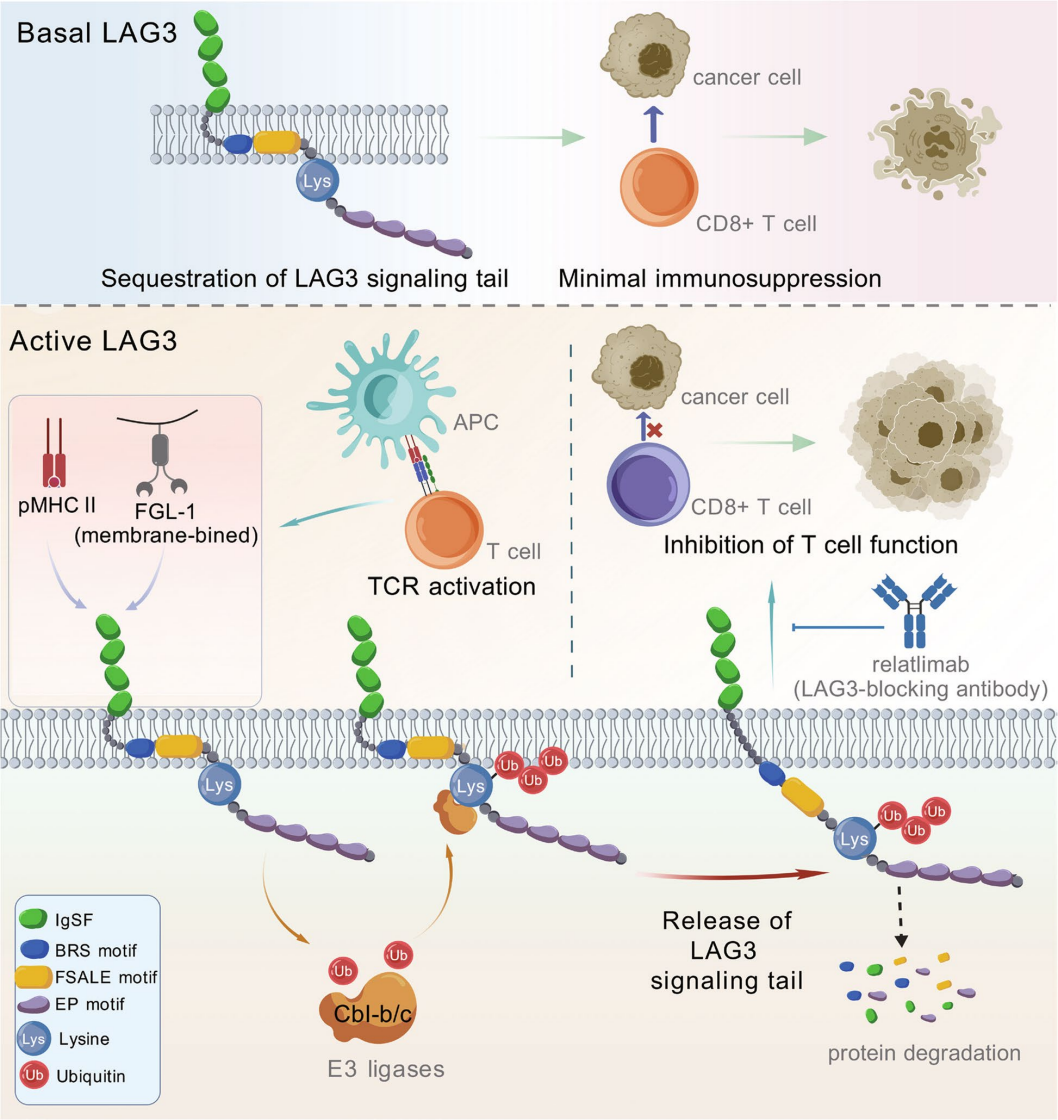
The basal state of LAG3 and its molecular activation mechanism upon ligand binding. In the basal state, the signaling tail of LAG3 is sequestered within the cell membrane, rendering it unable to exert immunosuppressive function. Upon stimulation by MHC-II on cancer cells or antigen-presenting cells (APCs), or by membrane-bound FGL1 (soluble FGL1 cannot induce this stimulation), T cells become activated. During this process, the E3 ligases c-Cbl or Cbl-b mediate polyubiquitination of LAG3 at K498. This polyubiquitination does not lead to LAG3 degradation but instead impede LAG3 membrane binding, releasing the BRS and FSALE motifs, the latter play critical roles in immune suppression. The LAG3-blocking antibody Relatlimab can inhibit both this polyubiquitination and the subsequent immunosuppressive process

LAG3 functions as a vital checkpoint receptor on immune cells, serving an essential purpose in controlling T cell activity and anti-tumor immunity [2]. Although its clinical significance has been recognized (e.g., through the approval of the LAG3-blocking antibody relatlimab [3]), the activation mechanism of LAG3 remains unclear. Unlike other checkpoint receptors such as PD-1 or CTLA-4, LAG3 doesn't possess standard inhibitory motifs within its cytoplasmic domain. Instead, it features conserved sequences—FSALE, KIEELE, and EP—whose functions in signaling processes remain debated.

The activation of immune receptors typically involves ligand-induced post-translational modifications (PTMs), which serve as major mechanisms for initiating or regulating immune receptor activity [4]. To investigate possible LAG3 PTMs under T cell activation, the researchers developed a co-culture model combining T cells with APC coculture and stimulated T cell receptors (TCRs) with superantigens to mimic physiological conditions. Using immunoprecipitation-mass spectrometry (IP-MS), they found that lysine 498 (K498) in the cytoplasmic tail of LAG3 undergoes ubiquitination during T cell activation, forming polyubiquitin chains with at least three ubiquitin molecules, while no such modification was detected in resting T cells. LAG3 is ubiquitinated solely at a conserved site, K498 (K490 in mice). The ubiquitination of this site is unaffected by the EP motif deletion, and mutating K498 is sufficient to completely block all LAG3 ubiquitination. Furthermore, LAG3 ubiquitination depends on ligand binding: MHC class II engagement robustly induces ubiquitination, membrane-bound FGL1 (FGL1-Fc) shows weaker induction, while free FGL1 (His-FGL1) fails to trigger ubiquitination. LAG3-blocking antibodies (e.g., relatlimab) or ligand-binding-deficient mutants completely suppress this modification.

Typically, ligand-induced receptor ubiquitination acts as a regulatory brake, facilitating receptor breakdown to curb excessive signaling [4]. To determine if ubiquitination of LAG3 influences its stability, the researchers examined protein levels of wild-type (LAG3^WT^) and ubiquitination-deficient (LAG3^KR^) LAG3 in human and mouse T cells during activation. Intracellular and surface expression levels remained comparable between LAG3^WT^ and LAG3^KR^. Treatment with the protein synthesis inhibitor cycloheximide (CHX) revealed similar half-lives for both variants, indicating that LAG3 ubiquitination doesn't regulate protein turnover.

IP-MS analysis showed that LAG3 ubiquitination primarily involves K48, K63, and K11-linked polyubiquitin chains. To determine the specific linkage type, the authors employed ubiquitin mutants that selectively permit formation of particular polyubiquitin chains while inhibiting others. The use of ubiquitination linkage-specific monoclonal antibodies has provided additional confirmation that LAG3 experiences mainly K63-linked polyubiquitination, with only minor K48-linked modification. Verification of K11-linked ubiquitination, however, wasn't possible because sufficiently specific antibodies for this linkage type are unavailable.

TurboID, an engineered biotin ligase, labels proximal proteins (~ 10 nm) when fused to a target protein. The biotinylated proteins can then be purified using streptavidin and analyzed by mass spectrometry or western blot. In their search for E3 ligases mediating LAG3 ubiquitination, researchers fused TurboID to LAG3's C-terminus for proximity labeling in activated T cells. Among over 80 ubiquitination-related labeled proteins, only Cbl family members (c-Cbl and Cbl-b) showed significant enrichment. Genetic knockout experiments demonstrated that while single deletion of either c-Cbl or Cbl-b failed to prevent LAG3 ubiquitination, dual knockout completely abolished it. Re-expression of either c-Cbl or Cbl-b in double-knockout cells restored LAG3 ubiquitination. Similarly, treatment with a Cbl small-molecule inhibitor significantly reduced LAG3 ubiquitination. In vitro ubiquitination assays further demonstrated that c-Cbl directly mediates LAG3 ubiquitination, establishing c-Cbl and Cbl-b as the primary E3 ligases regulating this process.

The cytoplasmic tail of LAG3 possesses a conserved motif rich in basic residues (BRS), situated adjacent to the FSALE signaling region—a crucial domain for LAG3-driven immunosuppression [5]. In other immune receptors such as CD3ε, CD28, and PD-L1, BRS motifs bind to membrane phospholipids, thereby sequestering signaling components. The authors proposed that LAG3-BRS similarly limits FSALE availability by tethering it to the membrane. To investigate how ubiquitination regulates LAG3 function, they employed aromatic fluorescence emission (AFE) and fluorescence resonance energy transfer (FRET) assays, demonstrating that both the membrane-proximal region of LAG3 (LAG3-NCR) and its complete cytoplasmic domain (LAG3-FL) specifically associate with negatively charged phospholipids in environments resembling cellular membranes. Nuclear magnetic resonance (NMR) spectroscopy analysis showed that phenylalanine 483 (F483) and leucine 486 (L486) located within the FSALE motif penetrate into the hydrophobic interior of the lipid bilayer, thereby stabilizing the membrane attachment. Ubiquitination at K498 substantially reduced membrane binding capacity, as shown by fluorescence polarization (FP) assays using a ubiquitin-fused LAG3-NCR (LAG3-NCR-Ub). In functional experiments, artificially blocking the membrane binding of LAG3 by inserting a 10-amino acid flexible linker peptide (LAG3^10AA^) between BRS and FSALE enhanced its immunosuppressive activity. Moreover, the functional impairment observed in the K498R mutant (LAG3^KR^) was completely restored through this intervention, verifying that LAG3's membrane-associated state is crucial for controlling its activity.

To investigate the immunological and potential clinical significance of LAG3 ubiquitination, the researchers analyzed T cell development and functionality in mice carrying the ubiquitination-deficient LAG3^KR^ variant. Although LAG3^KR^ had no impact on T cell development or peripheral maintenance, it substantially compromised the protein's immunosuppressive capacity. This was demonstrated by elevated cytokine (IL-2, IL-5, IL-17) secretion and a diminished ability to suppress CD4^+^ T cell proliferation following antigen stimulation. In MC38 colon carcinoma and B16 melanoma models, LAG3^KR^ mice phenocopied LAG3 knockouts, showing markedly inhibited tumor growth with 50% achieving complete tumor regression and enhanced infiltration of IFN-γ^+^ T cells in the tumor microenvironment (TME). Mechanistically, 7 clinically tested LAG3-blocking antibodies all suppressed LAG3 ubiquitination, and the inhibitory effect correlating positively with their therapeutic potency. Single-cell RNA sequencing analysis revealed that LAG3 and Cbl co-expression predominantly enriches in exhausted T cells (TEX) and correlates with poor prognosis. Notably, among patients receiving LAG3/PD-1 combination therapy, those who experienced clinical benefit showed a 51.7-times higher prevalence of LAG3^+^Cbl^+^ individuals compared to the non-responding group. In contrast, the LAG3-only cohort demonstrated merely a 6.5-fold increase. These findings validate that the co-expression of both Cbl and LAG3 can function as a predictive indicator for patient response to LAG3-targeted treatments.

In conclusion, the authors establish a model for LAG3 motif function. Under ligand-free conditions, LAG3 maintains a ubiquitination-independent tonic inhibitory effect via its constantly accessible EP motif, whereas the FSALE motif stays inactive and concealed within the membrane as a result of the upstream BRS motif's membrane association. Ligand binding initiates swift K498 ubiquitination within the KIEELE motif, releasing FSALE from membrane confinement and activating LAG3's immunosuppressive function. This model reveals a new paradigm for immune checkpoint regulation and opens avenues for next-generation immunotherapies.

In summary, this study is the first to elucidate the activation mechanism of LAG3 through non-degradative ubiquitination, which releases it from membrane sequestration, providing a new perspective on immune checkpoint regulation. LAG3 ubiquitination acts as both a dynamic biomarker for assessing antibody efficacy and a basis for precision patient selection in LAG3-targeted therapies. This discovery holds significant guiding value for optimizing cancer immunotherapy strategies, such as combining PD-1/LAG3 inhibitors.

## Data Availability

Not applicable.
